# The Sequence and Structure Determine the Function of Mature Human miRNAs

**DOI:** 10.1371/journal.pone.0151246

**Published:** 2016-03-31

**Authors:** Katarzyna Rolle, Monika Piwecka, Agnieszka Belter, Dariusz Wawrzyniak, Jaroslaw Jeleniewicz, Miroslawa Z. Barciszewska, Jan Barciszewski

**Affiliations:** Institute of Bioorganic Chemistry of the Polish Academy of Sciences, Noskowskiego 12/14, 61–704 Poznan, Poland; Mayo Clinic Arizona, UNITED STATES

## Abstract

Micro RNAs (miRNAs) (19–25 nucleotides in length) belong to the group of non-coding RNAs are the most abundant group of posttranscriptional regulators in multicellular organisms. They affect a gene expression by binding of fully or partially complementary sequences to the 3’-UTR of target mRNA. Furthermore, miRNAs present a mechanism by which genes with diverse functions on multiple pathways can be simultaneously regulated at the post-transcriptional level. However, little is known about the specific pathways through which miRNAs with specific sequence or structural motifs regulate the cellular processes. In this paper we showed the broad and deep characteristics of mature miRNAs according to their sequence and structural motifs. We investigated a distinct group of miRNAs characterized by the presence of specific sequence motifs, such as UGUGU, GU-repeats and purine/pyrimidine contents. Using computational function and pathway analysis of their targeted genes, we were able to observe the relevance of sequence and the type of targeted mRNAs. As the consequence of the sequence analysis we finally provide the comprehensive description of pathways, biological processes and proteins associated with the distinct group of characterized miRNAs. Here, we found that the specific group of miRNAs with UGUGU can activate the targets associated to the interferon induction pathway or pathways prominently observed during carcinogenesis. GU-rich miRNAs are prone to regulate mostly processes in neurogenesis, whereas purine/pyrimidine rich miRNAs could be involved rather in transport and/or degradation of RNAs. Additionally, we have also analyzed the simple sequence repeats (SSRs). Their variation within mature miRNAs might be critical for normal miRNA regular activity. Expansion or contraction of SSRs in mature miRNA might directly affect its mRNA interaction or even change the function of that distinct miRNA. Our results prove that due to the specific sequence features, these molecules can also be involved in well-defined cellular processes depending on their sequence contents. The pathway mapping and theoretical gene target identification allowed us to create a biological framework to show the relevance of the specific miRNAs in regulation the distinct type of targets.

## Introduction

Among all biological macromolecules, RNAs are one of the most structurally and functionally diverse molecular players within the cell. They fulfill many different functions such as coding and transferring genetic information, controlling various cellular processes, as well as catalyzing cleavage reactions, synthesis and ligations [1]. RNAs execute these roles through their variable and dynamic tertiary structures, which enable the RNA to interact specifically with themselves, with other RNAs, ligands and RNA-binding proteins [1]. miRNAs are a class of endogenous, non-coding small RNA, 19–25 nucleotides in length, that mediate the post-transcriptional regulation of mRNAs expression. They are evolutionary conserved, and control diverse biological processes as developmental timing, differentiation, cell proliferation, cancer or neurodegenerative disorders and apoptosis [2–9]. In the canonical pathway, the regulatory function of miRNAs is achieved primarily by binding target mRNAs at sites frequently located in the 3’ untranslated region (UTR). However, miRNA binding sequences have been identified also in the coding region and in 5’UTR [10–11]. miRNA activity requires its incorporation in a RNA-induced silencing complex (RISC). Here, a target recognition relies mainly on near-perfect complementarity of the mRNA with the miRNA “seed region”, 6–8 nucleotide-long sequence at the 5’ end of miRNA. Upon target binding, repression of gene expression is accomplished by suppressing translation, but some recent reports suggest that the major role of miRNAs is the reduction of target mRNA stability [2, 12–19]. The specificity of miRNA targeting in mammals is not restricted to the 5’ and 3’ ends of the seed region and Watson-Crick binding of the seed is not always sufficient for the repression of targets in mammals [20–21]. It has already been reported that the residues in positions 13–16 from the 5’ end of the miRNAs are involved in stabilization of miRNA-mRNA interactions, especially when base-pairing in the 5’ seed region is suboptimal [8, 20–22]. Unlike G and U residues, which can form wobble pairs (C/U or A/G), A residue can only pair with U residue of target mRNAs. It is known that sequence at 5’ end of miRNA determines the preferential strand selection and/or rejection in maturation process, between two strands of the same precursor duplex [21]. Therefore, pronounced occurrence of U at the 5’ end enhances the preference of the strand selection.

Thus, mature miRNA can control the expression of thousands of target mRNAs, and on the other hand, it is believed that a single mRNA is targeted by multiple miRNAs [20–23]. However, due to the current, incomplete understanding of the mechanism of miRNAs action beyond their biogenesis and target inhibition, the last process is somewhat unpredictable and often not as efficient as expected.

Recently, some non-canonical pathways of miRNA biogenesis have been proposed [24–28]. The analyses of data from next generation sequencing or microarrays, have revealed subclasses of miRNA species, derived from alternative biogenesis pathways. They only partially meet the classical definition, such as mirtrons—miRNAs derived from snoRNAs, from endogenous short-hairpin RNAs (endo-shRNAs) or tRNA precursors [27–28].

The broad analysis of the role of miRNA-target base-paring in regulation by miRNAs has been shown that this interaction is essential for miRNA specificity in regulatory processes [22, 28]. Although the strength of the interaction is one of the essential factors that can alter the stability of a dsRNA duplex, the contribution of the base-pairing potency to miRNA-target duplex stability remains still unclear. There was also already shown that the sequence of the particular class of miRNA is important for mRNA repression, e.g. in *Arabidopsis* stress-responsive miRNAs were found to be G/C rich [29]. This nucleotide content increases the miRNA-target duplex stability, which accelerates the stress response.

miRNA-mediated gene regulation and mRNA expression are undoubtedly dependent on both nucleic acid conformation and target recognition. We assumed then, if base-pairing potency could affect the functionality of the miRNA-mediated gene expression regulation, the full understanding of various biological functions of miRNA needs the knowledge about the sequence and structure of mRNA targets, miRNAs precursors but also of mature miRNAs is needed.

In this paper, we consistently studied across the mature human miRNAs: (i) the length distribution; (ii) characteristics of nucleotides content, their occupancy position, sequence–based motifs, like: UGUGU, GU-repeats and purine/pyrimidine contents; (iii) simple sequence repeats (SSRs). Additionally, we have also examined the structural tetranucleotide motifs (UUCG, GAAA, GCAA, GAGA, GUGA, GGAA, CUUG, UUUG) involved in stable hairpin formation and their propensity for regulation of specific group of targets. Using ModeRNA software, for the first time we have modeled the tertiary structure of mature miRNAs, suggesting that the miRNAs-target recognition can be not only sequence-, but also structure-related. Thus, in this paper we investigate the sequence features of mature miRNAs, convincing that the nature of these analysis can reveal the complexity of the miRNA molecular structures, what can give a hint for a genetic, including cancer, research. To predict the biological functions behind our sequence analysis results, we search for the corresponding pathways of the distinct group of miRNAs using DNA intelligent (DIANA) and Kyoto Encyclopedia of Genes and Genomes (KEGG) analyses. Accordingly, with DIANA-miRPath software we performed a computational target prediction combined with pathway analysis. Apart from the pathways analysis, the gene ontology including biological processes was also taken into account. The validity of the KEGG pathway clustering approach is further supported by the additional analysis with PANTHER classification system, that provided also the list of different protein classes being involved in the distinct processes and pathways. Based on these analysis we have found a link between the sequence content and pathways, biological processes and proteins associated with the distinct group of characterized miRNAs.

## Materials and Methods

### Library construction and clustering

All metazoan and human miRNAs sequences, available on 17^th^ October 2012, were downloaded from miRBASE version 19 in FASTA format (ftp://ftp.sanger.ac.uk/pub/mirbase/19.0/). The data were scanned for unique sequences of mature miRNAs. The molecules with identical sequences, but annotated under different names in the miRBASE, were removed. The cured library contained 2042 human miRNA sequences.

### Analysis of miRNA nucleotide sequences and SSR analysis

The analysis was done with the use of Phyton language, version 2.7.3. For each of the analyses, different scripts were prepared (S1 file). Whole sequence analysis was conducted on a specific number of sequences, to a single one accuracy.

For calculation of the monorepeats in a single miRNA sequences, the regular expressions language was used.

Di-, tri-, tetra- and pentanucleotide repeats were identified and localized by the software SSRIT (http://www.gramene.org/db/searches/ssrtool).

### Calculation SSR relative count

SSR relative count is the total repeats per miRNA on average. The calculated number of repeats were divided by the number of miRNAs is on each analyzed group (e.g. relative count for mononucleotide repeats 2326/2042 = 1.14).

### Functional analysis with DIANA miRPath

For the identification of the networks and pathways of the selected miRNAs we used DIANA miRPath (v2.0) software http://diana.imis.athena-innovation.gr) [30]. The prediction algorithm DNA Intelligent Analysis (DIANA) DIANA-microT-CDS (v5.0) was used for the identification of potential target RNAs from each cluster. A core analysis was performed to recognition of the most relevant miRNA targets, canonical pathways, biological functions and physiological processes from the interactions provided by the DIANA database. The identification of all the significantly targeted by the selected miRNA pathways was performed in the mode “Union of pathways”. The enrichment analysis and calculation of the significance levels (p-values) for each selected miRNA individually was performed. Fisher’s meta-analysis method was applying for calculation a merged p-value for each pathway. For the analysis a *posteriori* approach was used, where the statistical results showed the probability that the surveyed pathway is significantly enriched with gene targets of at least one selected miRNA. The significance for all the miRNA-mRNA pairs in a pathway were performed and calculated, followed by the combination into a merged P-value for each pathway. The results are reported and visualized as heat maps, and the pathways are clustered based on significance levels.

### Classification and pathway analysis with PANTHER System

The list of miRNAs with distinct sequence motif were subjected to TargetScanHuman program, release 7.0 (August 2015) analysis prior to obtain the full list of predicted targets for the specific group of miRNAs. The lists of targets were then uploaded to the PANTHER (protein annotation through evolutionary relationship) Classification System (http://pantherdb.org). By overrepresentation test the comparison of provided data set with a reference list in PANTHER database was performed. Ontology categories for over- and underrepresentation were determined statistically with Binominal distribution test.

### miRNA sequence alignment

ClustalW command line version of the multiplatform sequence alignment was used for comparison of distinct miRNAs sequences and other RNAs with previously, experimentally confirmed hairpin structures.

### Secondary structure analysis

Mfold program, version 3.5, was used to calculate the folding free energy in the conditions given by the program for all the sequences towards formation of hairpin structures (http://mfold.bioinfo.rpi.edu).

### miRNAs structure modeling

ModeRNA program (http://iimcb.genesilico.pl/moderna/) for miRNAs 3D modeling based on the experimentally confirmed structures (templates) was used [31]. The structure for RNAs were downloaded from PDB database. At least 80% of similarity was taken as a threshold with the sequences alignment. The PyMOL programm was applied as a tool for figures generation.

## Results

### The nucleotide composition in mature human miRNAs

The analysis of a length heterogeneity of 2042 sequences of human mature miRNAs resulted that number of nucleotides is a discrete random variable, which ranges from 16–27. miRNAs sequences with 21 (12%) and 23 (14%) nucleotides are most abundant, nevertheless miRNAs with 22 nucleotides in length are predominant (47%) (S1 Fig). These results corroborate with the recently published observations [32]. The sequence length analysis of human mature miRNAs showed that the length heterogeneity is related to some biological factors such as evolution conservations or miRNA’s regulatory mechanism [32]. It has been also established that miRNAs that regulate cancer-associated targets (oncogenes/tumor suppressors) show stable lengths of 22 nucleotides, whereas longer miRNAs tend to regulate more genes [32].

**Fig 1:**
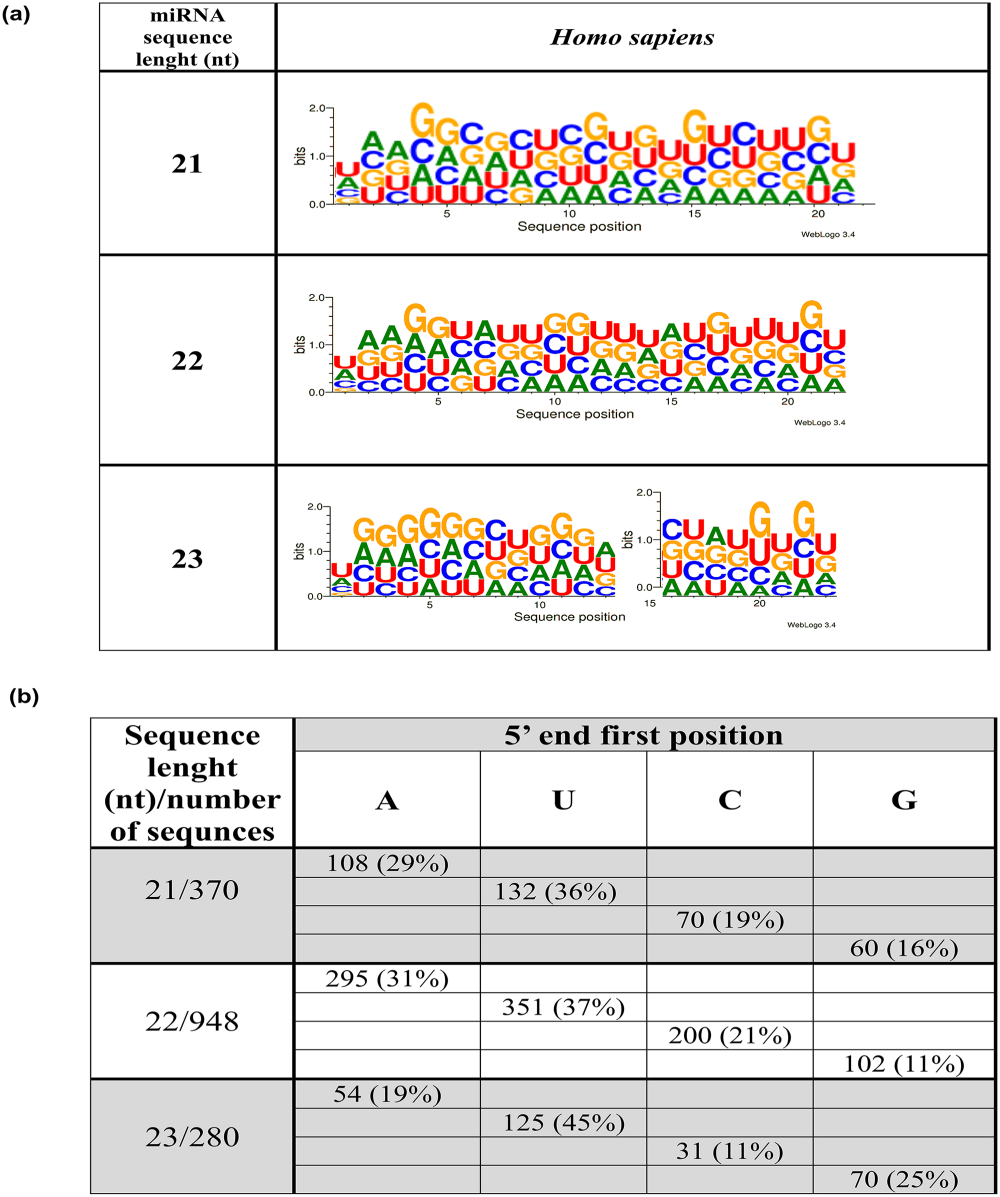
Nucleotide occupancy position in human mature miRNAs. A Web logo with the distinct nucleotides at all positions of the mature miRNA sequences. B. Nucleotides representation at the first position of miRNAs mature strand at 5’ and 3’ end, in context to their length. The number under the name of nucleotide (e.g. 108 for A) reflects 108 sequences with A as a first nucleoside at 5’ end, and 27 at 3’, respectively.

Furthermore we analyzed the nucleotides content in human mature miRNAs, what showed inequality of base presence. It showed a prevalence of guanosine and uridine at 29 and 26%, and lower levels of adenosine (23%) and cytidine (22%), respectively. This observations is also consistent with the previously shown results [32]. Although the most abundant nucleotide in miRNAs is guanosine, the first position at 5’ end is more frequently occupied by the uridine (35%) (Fig 1a and 1b). Furthermore, the second and third position are usually occupied by adenosine, making the 5’end (nucleotides position- 1–3) mostly UAA-rich. The 5’end for the most represented lengths of mature miRNAs: 21 nad 22 nt long is UAA-rich, what is shown on Fig 1a. miRNAs with 23 nt in length at the 5’ end show mostly UGG sequence. The seed sequence which is considered as position 2–8 is basically G-rich for 21 and 23 nt long miRNAs, whereas for 22 nt long- U/A rich.

However, one can notice, that A nucleotide at 2^nd^ and 3^rd^ positions is only slightly overrepresented than G. (Fig 1a and 1b). It has been also observed that the presence of A residue in the targeted mRNA, from position 11 of the 5’ end of a miRNA, has a great impact on the improvement of the miRNAs binding to the specific target [20, 33]. This strengthen of the interaction is observed, although it was shown, that the exact base-pairing between the A residue and the nucleotide at position 1 of the 5’end of the miRNA is not necessary [20, 22]. The strongest representation of the U residue in the first and the last position of miRNAs sequence was observed not only for human, but also five other mammalian species, such as *Pongo pygmaeus*, *Pan troglodytes*, *Macaca mulatta*, *Mus musculus* and *Rattus norvegicus*. This observation suggests that both ends, more likely than just the 5’ end, are also involved in target recognition [34].

Based on a single position analysis of the whole sequences, we have also looked at the purines or pyrimidines tracts in mature miRNAs. We assumed the threshold for purine/pyrimidine rich sequences over 70% for G, A and C,U, respectively. Based on that criterion we found 141 CU-rich and 187 AG-rich sequences. We found: miR-4290, miR-1281, miR-4716-5p, miR-483-3p and miR-877-3p containing almost only UC nucleotides and miR-6124, miR-483-5p, miR-4271, miR-4644, miR-4716-3p and miR-1234-5p purine-rich. In the Table 1 we showed only highly purine/pyrimidine rich sequences, were the level of the investigated nucleotides is over 90%.

**Table 1:** Pyrimidines- and purines-rich sequences of miRNAs. The table shows the content of the pyrimidines or purines nucleotides according to the sequence of miRNAs. The total contents of purines or pyrimidines of the miRNAs presented in the table is above 90%.

miRNA	Sequence	Pyrimidine/purine content [%]	Sequence lenght
**Pyrimidines-rich sequences**			
hsa-miR-4290	UGCCCUCCUUUCUUCCCUC	95	19
hsa-miR-1281	UCGCCUCCUCCUCUCCC	94	17
hsa-miR-4716-5p	UCCAUGUUUCCUUCCCCCUUCU	91	22
hsa-miR-483-3p	UCACUCCUCUCCUCCCGUCUU	90	21
hsa-miR-877-3p	UCCUCUUCUCCCUCCUCCCAG	90	21
**Purines-rich sequences**			
hsa-miR-6124	GGGAAAAGGAAGGGGGAGGA	100	20
hsa-miR-4271	GGGGGAAGAAAAGGUGGGG	95	22
hsa-miR-483-5p	AAGACGGGAGGAAAGAAGGGAG	95	22
hsa-miR-4644	UGGAGAGAGAAAAGAGACAGAAG	91	23
hsa-miR-4716-3p	AAGGGGGAAGGAAACAUGGAGA	91	22
hsa-miR-1234-5p	GGGGGGGGGGGGGGGGGGCCG	90	21

**Fig 2:**
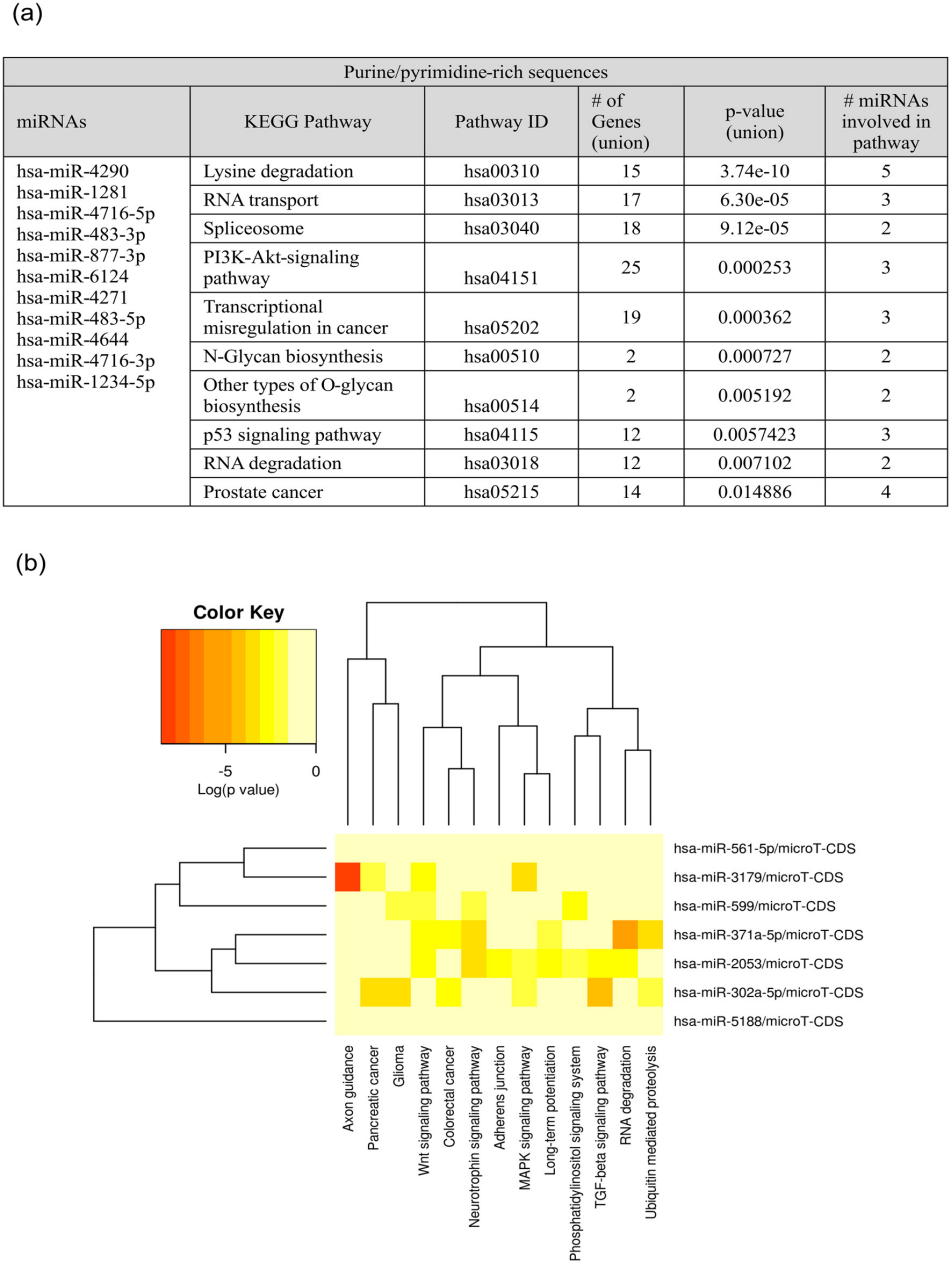
The KEGG pathway analysis for pyrimidine/purine (P/P)-rich miRNAs. A. The table illustrating the: list of P/P-rich miRNAs (first column); the IDs and KEGG pathways names (second and third column); the number of genes and P/P-rich miRNAs involved associated with the pathways (fourth and sixth column). P-value was given in fifth column as a result of statistical analysis. P-value threshold is considered 0.05. B. P/P-rich miRNAs in predicted pathway heat map. Significant miRNA-pathway interaction p<0.001.

The functional analysis of the cellular pathways analysis showed that the purine/pyrimidine-rich sequences show the involvement mostly in lysine degradation, RNA transport and spliceosome (Fig 2a, S1 Table). The search generated a rank-ordered list of KEGG and PANTHER pathways, issuing statistical significance based on p-values. Examining the pathways affected by the distinct group of miRNAs we highlighted pathways associated with different physiological processes connected to the specified group of miRNAs. The heat map indicates the high enrichment of the e.g. miR-483 in RNA degradation, miR-4271 in lysine degradation or miR-4716 in spliceosome (Fig 2b). The list of proteins involved in the pathways and processes associated with the purine/pyrimidine-rich miRNAs also corroborate with these results (S1 Table). The membrane trafficking regulatory, membrane traffic or nucleic acid binding proteins are the most common proteins being a main players in binding and RNA transport processes (S1b Table). Apart from the targeting the distinct group of mRNAs, polypyrimidine- tracts could allow possibly for the interaction of miRNAs with other proteins, such as PTB (polypyrymidine tract-binding protein), preventing the typical regulatory action of miRNAs [35]. On the other hand, such miRNAs can interact with Kozak sequence- purine-rich motif, similar to prokaryotic Shine-Dalgarno sequence [36–37]. Recently, the novel mechanism for post-transcriptional control of gene expression was shown [38]. It involves the formation of an intermolecular G-quadruplex between small RNA and mRNA, where a four stranded RNA structure is formed through direct RNA-RNA dimerization. This is hint to suppose, that the G-rich miRNAs could be also regulate the gene expression via this kind of mechanism.

### Simple sequence repeats (SSR) analysis

Regardless the extensive research, there are only limited data about the nucleotide composition, especially in terms of the nucleotide repeats in mature miRNAs. This analysis could be beneficial, since the SSRs can be potentially used for genetic maps construction, as a genetic markers, but also in the linkage association research or phylogenetics and population genetics studies [39–44]. It was also shown, that SSRs could serve for a target genes involved in diseases, for evolution history research or finally, in the execution of the paternity tests [45–48].

Based on the nucleotide sequence analysis, we performed SSR monitoring. We searched in human miRNAs for the occurrence and nature of SSRs. The most important standard for the identification of SSRs in a given sequence is the definition of the minimum repeat number. The minimum repeat units in track is annotated as the valid SSR tract. In order to detect various repeats in mature miRNAs, and taking into account the length of the molecules, we considered minimum three repeats. Consequently, three minimum repeats were used for the survey of SSRs in pre-miRNA studies [49]. Among the analyzed mature miRNA sequences we were able to define: mono-, di-, tri-, tetra- and pentanucleotide repeats.

Mononucleotide repeats poly (U) and poly (G) were the predominant repeats in all of the analyzed mature miRNAs (Table 2). The longest mononucleotide repeats observed, was the 18 nucleotides SSR poly (G). For poly (U), (A) and (C) repeats we were able to find maximum 10 nucleotides long, both for “U” and “A” SSR and 7 for “C”, respectively. Dinucleotides were the second most common repeats in mature miRNAs (Table 2). We have found that GU/UG were also predominant for human miRNAs (Table 2). To get comprehensive view on specified miRNAs, we established the list GU/UG-rich miRNAs (Table 3). The less frequent, with almost the same relative count, were the AG/GA and AU/UA repeats (Table 2). CG/GC repeats in mature miRNAs are relatively rare (Table 2). The most frequent dinucleotide repeat we have found within the sequence of has-miR-1277-5p-1, was (AU) motif, repeated 7 times.

**Table 2:** Occurrence and relative count of SSRs in mature miRNAs. Occurrence and relative count of all SSRs in mature miRNAs:mononucleotides, dinucleotides and the list of the longest tetra-and pentanucleotides.

SSRs	Mono	Di	Tri	Tetra	Penta	Total
**Total numbers of repeat**	2326 (1.14)	324 (0.16)	43 (0.02)	3 (0.001)	4 (0.002)	2700 (1.32)
**Repeat type and number**	**A**: 462 (0.23) **U**: 569 (0.28) **G**: 802 (0.40) **C**: 493 (0.24)	**AG/GA**: 66 (0.03) **GU/UG**: 83 (0.04) **AC/CA**: 45 (0.02) **CU/UC**: 67 (0.03) **AU/UA**: 47 (0.02) **CG/GC**: 16 (0.008)	See details in S2 Table	**(AGGA)**_**3**_	**(UGGGC)**_**4**_	2700 (1.32)

**Table 3:** The list of GU-rich miRNAs. GU-tracts are marked in bold among the sequence.

miRNA	Sequence
miR-574-5p	UGA**GUGUGUGUGUGU**GA**GUGUGU**
miR-941	CACCCGGCU**GUGU**GCACAU**GU**GC
miR-3149	UUU**GU**AUGGAUAU**GUGUGUGU**AU
miR-1238-5p	**GU**GA**GU**GGGAGCCCCA**GUGUGU**G
miR-545-3p	UCAGCAAACAUUUAU**UGUGU**GC
miR-2278	GAGAGCA**GUGUGUGU**UGCCUGG
miR-3148	UGGAAAAAACUG**GUGUGU**GCUU
let-7b-5p	UGAG**GU**A**GU**AG**GU**U**GUGU**G**GU**U
miR-493-3p	UGAAG**GU**CUACU**GUGU**GCCAGG
miR-1180	UUUCCGGCUCGC**GU**GG**GUGUGU**
miR-539-5p	GGAGAAAUUAUCCUUG**GUGUGU**
miR-32-3p	CAAUUUA**GUGUGUGU**GAUAUUU
miR-206	UGGAAU**GU**AAGGAA**GUGUGU**GG
miR-1299	UUCUGGAAUUC**UGUGUG**AGGGA
miR-3911	U**GUGU**GGAUCCUGGAGGAGGCA
miR-297	AUGUAU**GUGU**GCAU**GU**GCAUG
miR-610	UGAGCUAAAU**GUGU**GCUGGGA
miR-1228-5p	**GU**GGGCGGGGGCAG**GUGUGU**G
miR-595	GAA**GUGU**GCC**GU**G**GUGUGU**CU
miR-4455	AGG**GUGUGUGUGU**UUUU
miR-3650	AG**GUGUGU**CU**GU**AGA**GU**CC
miR-147a	**GUGUGU**GGAAAUGCUUCUGC
miR-660-3p	ACCUCCU**GUGU**GCAUGGAUUA

Interestingly, most of the trinucleotide repeats contain base “U” and “G”, although it can be noticed that this type of SSR is less represented. AGU, GGU and UGU SSR were not observed in humans mature miRNAs sequences, whereas GUG motif was observed 6 times. The detailed data about trinucleotide repeats are given in S2 Table. Mature human miRNAs with the longest trinucelotide repeat- (AGG)_4_ is hsa-miR-4298-1.

Although, in the mature miRNAs tetranucleotide and pentanucleotide repetas were less expected to detect, however we were still able to find such SSRs type (Table 2). Interesting example of mature miRNA, with pentarepeats, is hsa-miR-3620-5p-1, in which we identified (UGGGC)_4_ (Table 2).

Until now, there is only a single study focused on SSRs in short sequences, such as pre-miRNAs [49]. However, since miRNAs are functional molecules, we decided to monitor the SSRs in mature miRNAs. For this group of molecules, we have noticed the same tendency that has been already observed for pre-miRNAs. Among the majority of the analyzed miRNAs, poly (A/U) repeats were more frequent than poly (G/C) repeats. Having converted this data into genome level, this observation, both among the previous pre-miRNAs analysis and our mature miRNAs studies, is similar to primate genome [50]. Mononucleotide and dinucleotide repeats were significantly predominant, which was similar to pre-miRNAs and to that of introns, in which majority of SSRs were also mono- and dinucleotides, whereas tri-, tetra-, penta- and hexanucleotide repeats were relatively rare. It has been reported that (GT)_n_ is the most predominant dinucleotide repeat motif in animal and invertebrates. Additionally, similarly to pre-miRNAs sequences, the most abundant repeats were (GU)_n_/(UG)_n_ as well. Thus, one could assume that SSRs, both in pre-miRNAs and miRNAs, might have a functional meaning. They can be consider for providing a molecular basis for organization of pre-miRNAs *in vivo* or rapid mature miRNAs maturation. SSRs changes within the mature miRNAs sequences, similarly to pre-miRNAs might have a critical impact on the normal miRNA regulation activity and the variations of SSRs in mature miRNAs can influence theirs direct mRNA target interaction or even alter the function of that distinct miRNA.

**Fig 3:**
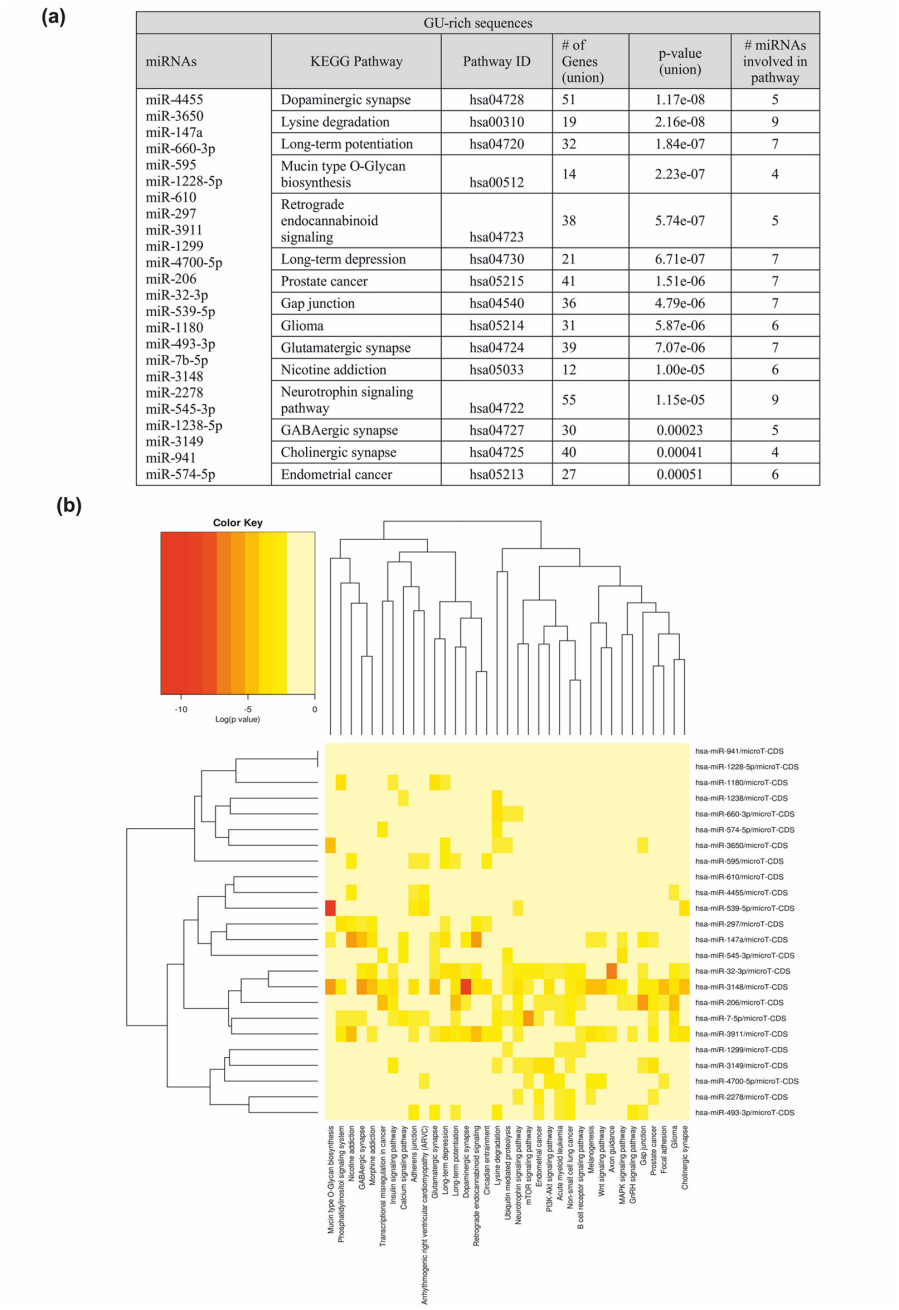
The KEGG pathway analysis for GU-rich miRNAs. A. The table illustrating the: list of GU-rich miRNAs (first column); the IDs and KEGG pathways names (second and third column); the number of genes and GU-rich miRNAs involved associated with the pathways (fourth and sixth column). P-value was given in fifth column as a result of statistical analysis. P-value threshold is considered 0.05. B. GU-rich miRNAs in predicted pathway heat map. Significant miRNA-pathway interaction p<0.001.

Additionally, our analysis showed that miRNAs enriched with the particular sequence motif are predicted to target different pathways. The pathways associated with GU-rich miRNAs as the most significant indicate a “dopaminergic synapse”, “lysine degradation” and “long-term potentiation”. The more intense red color in a heat map indicates higher probability that a specific pathway is significantly enriched with target genes for a certain miRNA (Fig 3). This analysis also revealed that the GU-rich miRNAs more likely are involved in the neurological processes such as “Opioid proopiomelanocortin pathway” or “Axon guidance mediated by netrin” (Fig 3, S3 Table). The list of the most important proteins involved in the processes with the participation of GU-rich miRNAs covers among others proteins of voltage-gated sodium channel, sodium channel, but also SNARE proteins, which the primary role is to mediate lysosome formation, particularly in the presynaptic membrane in neurons (S3 Table). Although AU-rich elements (AREs) are very abundant in the 3’UTRs of many different mammalian mRNAs with unstable structure, the presence and function of GU-rich elements (GREs) are still poorly understood. There was found that through genome-wide analysis at least 5% of human genes contain GREs in their 3’UTR. The functional over-representation of it is assigned for the genes involved in transcription, nucleic acid metabolism, developmental processes and neurogenesis [51]. Until now, there is no report showing the importance of the GU-rich miRNAs in specific processes. However, in our global sequence analysis of miRNAs, we demonstrate, that this type of miRNAs target also mRNAs involved in biological processes such as mRNA 3’- end processing, mRNA transcription or nervous system development (S3 Table). On the other hand, it was shown that GREs are the targets for at least one RNA-binding protein: CUG-binding protein 1 (CUG-BP1)–the member of the highly conserved CELF family of RNA-binding proteins that are post-transcriptional regulators of deadenylation, mRNA decay, translation and pre-mRNA processing [52–57]. It is possible CUG-BP1 could also bind directly GU-rich miRNAs and affect the protein expression. This needs, however, further investigation.

### The identification of interferon induction motif (IIM)

Going deeper into the sequence analysis within the sequences of mature miRNAs, we identified 5’-UGUGU-3’ motif, which is known for stimulation of innate immune response. We have found 50 of mature miRNAs within that motif (Table 4). That feature of the UGUGU sequence was first shown for siRNAs as extremely important, as it may cause variety of non-specific side effects, including stimulation of interferon and cytokine production, global shutdown of protein synthesis or nonspecific degradation of mRNAs [58–59]. The miRNA, containing interferon stimulation motifs, can induce itself one or more undesirable effects, such as proliferation blockage, differentiation or apoptosis of cancer cells, or it can even serve as potent immunomodulatory agent [58–59].

**Table 4:** Selected human miRNAs contained immunostimulatory motifs. The table presents the miRNA sequences with at least one UGUGU motif (highlighted in bold).

miRNA	Sequence (5’-3’)	miRNA	Sequence (5’-3’)
miR-3682-5p	CUACUUCUACC**UGUGU**UAUCAU	miR-539-5p	GGAGAAAUUAUCCUUGG**UGUGU**
miR-2278	GAGAGCAG**UGUGU**GUUGCCUGG	miR-1180-3p	UUUCCGGCUCGCGUGGG**UGUGU**
miR-1238-5p	GUGAGUGGGAGCCCCAG**UGUGU**G	miR-450a-5p	UUUUGCGA**UGUGU**UCCUAAUAU
miR-3149	UUGUAUGGAUA**UGUGU**GUGUAU	miR-493-3p	UGAAGGUCUAC**UGUGU**GCCAGG
let-7b-5p	UGAGGUAGUAGGU**UGUGU**GGUU	miR-4669	**UGUGU**CCGGGAAGUGGAGGAGG
miR-4455	AGGG**UGUGU**GUGUUUUU	miR-4753-3p	UUCUCUUUCUUUAGCCU**UGUGU**
miR-4717-5p	UAGGCCACAGCCACCCA**UGUGU**	miR-624-5p	UAGUACCAGUACCU**UGUGU**UCA
miR-573	CUGAAGUGA**UGUGU**AACUGAUCAG	miR-3657	**UGUGU**CCCAUUAUUGGUGAUU
miR-660-3p	ACCUCC**UGUGU**GCAUGGAUUA	miR-3148	UGGAAAAAACUGG**UGUGU**GCUU
miR-378a-5p	CUCCUGACUCCAGGUCC**UGUGU**	miR-3177-5p	**UGUGU**ACACACGUGCCAGGCGCU
miR-599	GU**UGUGU**CAGUUUAUCAAAC	miR-581	UCU**UGUGU**UCUCUAGAUCAGU
miR-1226-3p	UCACCAGCCC**UGUGU**UCCCUAG	miR-642b-5p	GGUUCCCUCUCCAAA**UGUGU**CU
miR-1270	CUGGAGAUAUGGAAGAGC**UGUGU**	miR-147a	G**UGUGU**GGAAAUGCUUCUGC
miR-610	UGAGCUAAA**UGUGU**GCUGGGA	miR-592	U**UGUGU**CAAUAUGCGAUGAUGU
miR-3911	**UGUGU**GGAUCCUGGAGGAGGCA	miR-545-3p	UCAGCAAACAUUUAU**UGUGU**GC
miR-3650	AGG**UGUGU**CUGUAGAGUCC	miR-1228-5p	GUGGGCGGGGGCAGG**UGUGU**G
miR-4789-5p	GUAUACACCUGAUA**UGUGU**AUG	iR-642a-5p	GUCCCUCUCCAAA**UGUGU**CUUG
miR-330-5p	UCUCUGGGCC**UGUGU**CUUAGGC	miR-3152-3p	**UGUGU**UAGAAUAGGGGCAAUAA
miR-941	CACCCGGC**UGUGU**GCACAUGUGC	miR-32-3p	CAAUUUAG**UGUGU**GUGAUAUUU
miR-597	**UGUGU**CACUCGAUGACCACUGU	miR-1299	UUCUGGAAUUC**UGUGU**GAGGGA
miR-5580-5p	UGCUGGCUCAUUUCAUA**UGUGU**	miR-297	AUGUA**UGUGU**GCAUGUGCAUG
miR-4700-5p	UCUGGGGAUGAGGACAG**UGUGU**	miR-187-3p	UCGUGUCU**UGUGU**UGCAGCCGG
miR-892a	CAC**UGUGU**CCUUUCUGCGUAG	miR-5010-3p	UUU**UGUGU**CUCCCAUUCCCCAG
miR-206	UGGAAUGUAAGGAAG**UGUGU**GG	miR-574-5p	UGAG**UGUGUGUGUGU**GAG**UGUGU**
miR-595	GAAGUGUGCCGUGG**UGUGU**C	miR-649	AAACC**UGUGU**UGUUCAAGAGUC

**Fig 4:**
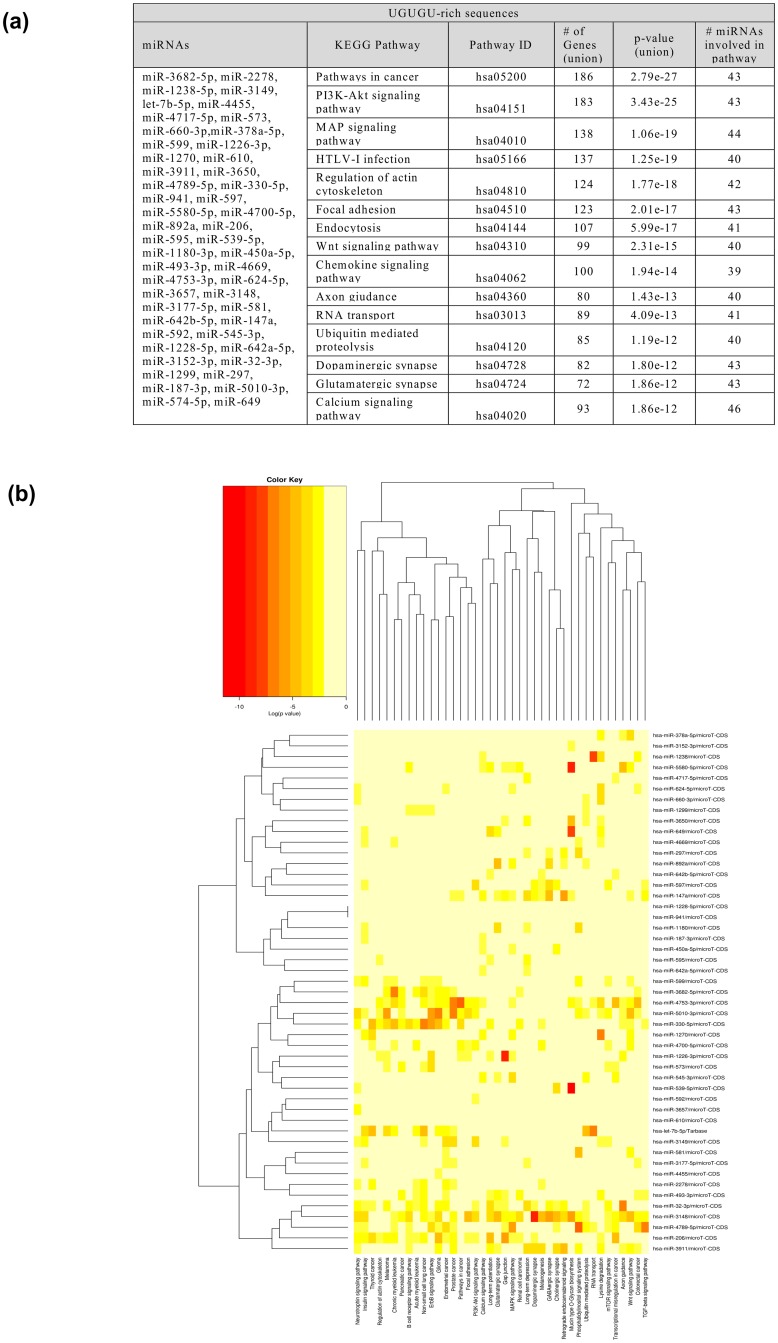
The KEGG pathway analysis for interferon induction motif contained-(IIM) miRNAs. A. The table illustrating the: list of IIM-contained miRNAs (first column); the IDs and KEGG pathways names (second and third column); the number of genes and IIM-rich miRNAs involved associated with the pathways (fourth and sixth column). P-value was given in fifth column as a result of statistical analysis. P-value threshold is considered 0.05. B. IIM-rich miRNAs in predicted pathway heat map. Significant miRNA-pathway interaction p<0.001.

We have found, that apart from the potential of these group of miRNAs to possible direct activation of interferon response, they also target particular mRNAs involved in specific pathways and cellular processes. Analysis for UGUGU-rich miRNAs ranked at the top of the list following pathways: pathways in cancer, PI3K-Akt signaling, MAP-signaling and hedgehog signaling pathways (Fig 4, S4 Table). Our analysis showed that these type of miRNAs is involved among others in processes such as induction of apoptosis, immune system process, immune response or macrophage activation (S4 Table). Due to the KEGG and PANTHER analyses of miRNAs involvement in this type of biological processes, it appears, that also for IIM-rich miRNAs we could expect the direct recognition of the mRNAs involved in PI3K-Akt signaling and MAP signaling pathways (Fig 4a, S4 Table). The potential involvement of IIM-contained miRNAs in these pathways are also supported by the list of protein class provided by the PANTHER analysis (S4 Table). It was shown that positioning of this 5′-UGUGU-3′ motif especially at the 5′- end of the sense strand of siRNAs results in a rapid and enhanced induction of type I IFN. Rapid production of IFN –β involves thus activation of signaling cascades governed by effectors that are intermediates in the JAK/STAT, mitogen-activated kinase (MAPK) and phosphatidylinositol 3-kinase (PI3K) pathways [60–61].

This results indicates that IIM-rich miRNAs, similarly to the cancer-associated miRNAs (CA-miRNAs) are closely connected to cancer [62]. The one of the most prominently observed pathway for these class of IIM- miRNAs- pathways in cancer is highly represented by the gene targets for IIM-miRNAs. This kind of pathway clearly demonstrated the involvement into biological capabilities such as evading of apoptosis, block of differentiation, unlimited replication, increased angiogenesis, sustained ability for invasion and metastasis in malignant transformation [62]. Mitogen –activated protein kinase (MAPK) pathway functions also as integrating signals that affect proliferation, differentiation, survival and migration. The results indicates, that the IIM-rich miRNAs could also be a promising clinical targets for cancer through the MAPK pathway. The phosphatidylinositol 3-kinase(PI3K)-Akt signaling pathway is activated by many types of cellular stimuli or toxic agents and it is involved in regulation of basic physiological cellular functions such as transcription, translation, proliferation, growth or survival [63]. There was shown that serine/threonine kinase Akt/PKB plays significant role in this pathway. Moreover, a impaired activation of the PI3K-Akt pathway has been associated with the development of different types of diseases such as diabetes, mellitus, autoimmunity, and finally- cancer [64].

### The characteristic of stem-loop hairpin structure of mature human miRNAs

It is already known that over 50% of the hairpins predicted for the human 16S and 23S rRNA have tetranucleotides loops [65]. The tetraloops are thought to fulfill a variety of functions, including recognition elements for interactions with proteins and other RNAs. They can regulate the activity of a biological system by shifting the equilibrium between alternate structures [66]. It was observed, with the use of the NMR methods that small stem-loops can exist, in solution, in equilibrium with duplex forms [67]. About 70% of these tetraloops have the consensus loop sequences (GNRA) or UNCG (where N = A,C,G or U; and R = A or G. It has been shown that RNA hairpins within these sequences form unusually stable loop conformation [65–67].

With Mfold algorithm, we found 1431 (70%) sequences of human miRNAs, able to fold into the hairpin structure, whereas 588 sequences did not show such a propensity. The minimal free energy of these structures (*ΔG*) falls in the range from -0.1 to -11.1 kcal//mol, however, the most widely represented structures indicate the energy levels from -0.4 to -3.3 kcal/mol [68]. In order to gain better insight into the potential secondary structure of mature miRNAs, we have also analyzed a possibility of the hairpin stem-loop formation. Our calculation revealed that the most frequently motif occurred in miRNAs, is the four nucleotides motif (tetraloop), found in 433 sequences of mature RNAs. Other hairpin-loop of miRNA sequences are: 413, 283 and 114 for three-, five- and six-nucleotides loops, respectively.

Among tetraloops, we specified sequences: UUCG, GAAA, GCAA, GAGA, GUGA, GGAA, CUUG, UUUG—involved in the hairpin loop formation. Many sequences of mature miRNAs can fold exactly into the hairpin loop, with the nucleotides of interest located in the loop, containing minimum 3 base pairs in the stem additionally (Table 5).

**Table 5:** Occurrence of tetranucleotides motifs connected with hairpin formation in human miRNA sequences. The all miRNAs with the ability to form secondary structure were subjected to the analysis. The searching was performed according to: first—motifs present in loop of the predicted hairpins, second: motifs present in the loop where at least 3 base pair were predicted in the stem and finally-where at least 3 base pair and additionally C-G as a closing pair in the stem were predicted.

Motif	Frequency		
	Sequences		
	Hairpin-formation	3 base-pairs in stem	3 base-pairs and C-G as a closing pair in stem
**GGAA**	25	21	8
**CUUG**	10	8	4
**GCAA**	13	10	2
**GUGA**	23	17	0
**GAAA**	19	17	3
**GAGA**	32	18	1
**UUCG**	5	5	2
**UUUU**	28	21	12
**UUUG**	24	22	6
**Total**	179	139	38

We found that 179 miRNAs can form stable hairpin structure, with the 4-nt motifs (Table 5). The most represented motif in human miRNA sequences is GAGA, although GGAA, GAAA or UUUU motifs are also abundant. UUUU motif in the loop, together with the CG as a closing pair in the stem, is most widely represented among the mature human miRNAs (Table 5).

The observation that miRNA can fold into the secondary structure is consistent also with previous reports, that plant miRNA can form secondary structure with free energy of: -9.3 to +1.5 kcal/mol [69]. It directly supports the observation that given miRNAs differ dramatically in terms of the half-life. Thus, the knotty secondary structures of miRNAs makes them nucleases resistant, implicating their long surveillance in the cell. The higher order structure of miRNA can play a crucial role in conformational changes during miRNA-mRNA interactions. This could modulate the pairing and also explain the different degree of genetic regulation for the specific miRNA. The secondary structure could be also extremely important in the mechanism by which sequences for some miRNAs are selected, what can modulate its affinity with their mRNA targets. It can also provide the specificity of miRNAs-mRNA interaction what could be achieved by the presence of miRNA secondary structure, by precluding the possibility of binding of other miRNAs and genomic RNAs with complementary sequences.

Although the mature miRNAs are generally considered as single stranded, there are very few reports suggesting a self-complementarity in mature miRNAs, that has already been observed in more than 50% of mature miRNAs. They are prone to form hairpins and/or homo-duplexes in solution [38, 57–58]. NMR studies have shown that hsa-miR-520h mature strand can fold into hairpin structure or into self-complementary homo-duplex, in higher concentration [70].

**Fig 5:**
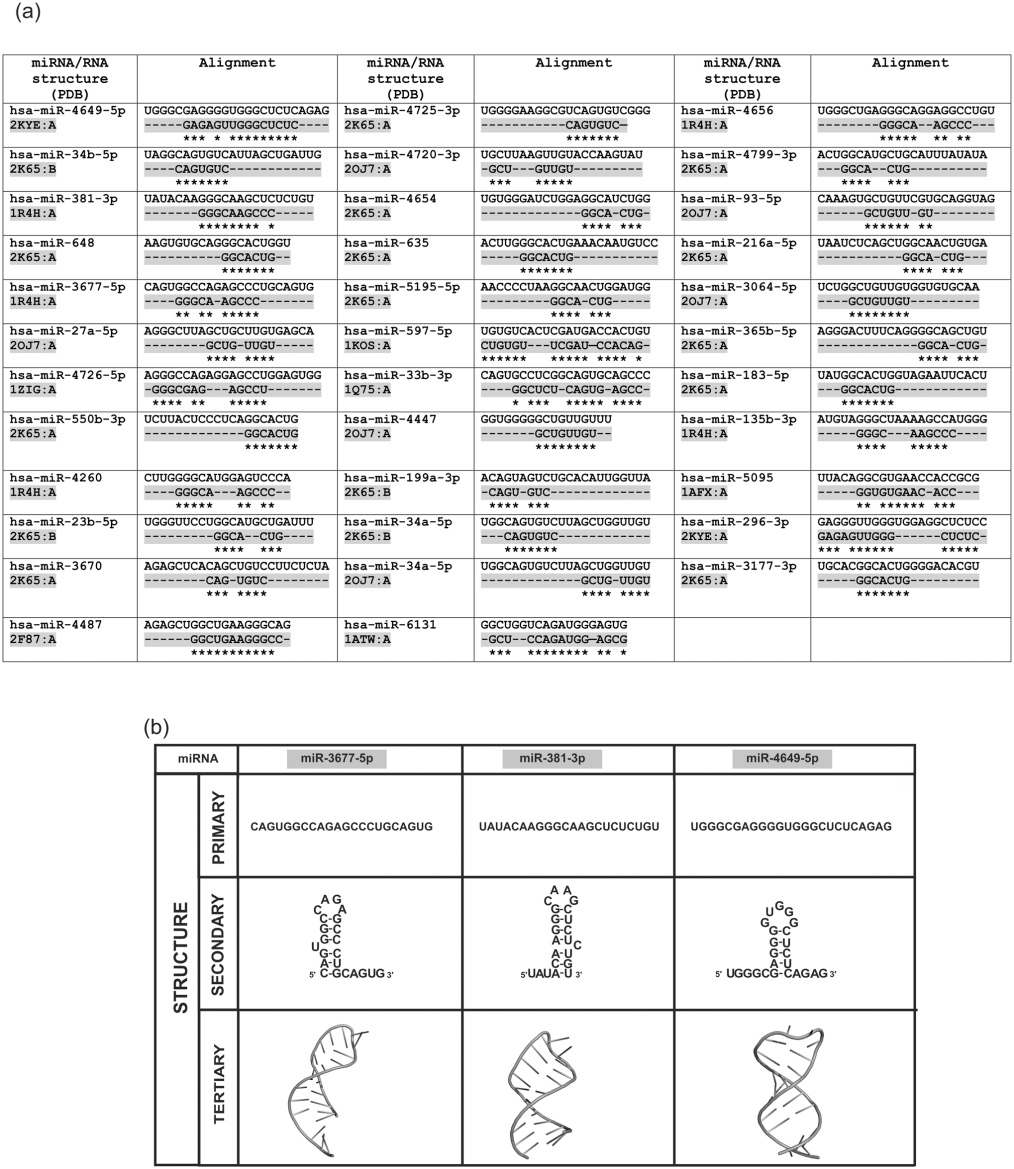
Tertiary structure of miRNAs as a result of ModeRNA modeling. The homological comparison of miRNAs to the known, experimentally confirmed short RNA sequences was performed in the three main steps: sequence analysis (primary structure), secondary structure folding and tertiary structure modeling. A. Sequence alignment of miRNAs to the experimentally confirmed short RNAs. B. Three selected miRNAs modeled in ModeRNA program with high similarity to the short RNA, which structures are deposited in PDB.

Next, we used the ModeRNA program for modelling of miRNA 3D structure, based on templates of related molecule. To find tertiary structures, we searched the PDB database for experimentally confirmed (X-ray, NMR or CD), about 24 nucleotides long RNAs. We have found 168 sequences, which were aligned to human mature miRNA sequences. For further analysis, we only took into account sequences with at least 80% similarity, since only that level provides reliable, homology modeling. The alignment showed 35 miRNAs that were further processed with ModeRNA server (Fig 5a). Positions with identical nucleotides were fixed, whereas remaining positions were modeled by the program. The best matched results we obtained include miR- 381-3p with 1R4H RNA, miR-4649-5p with 2KYE and miR- 3677-5p with 1Q8N RNA (Fig 5b). Due to the homology modeling, ModeRNA analysis makes our calculation and secondary structure prediction more accurate and give strong support for miRNAs hairpin loop formation hypothesis.

Our CD, NMR and enzymatic probing data for some miRNAs also prove that miRNAs have the intrinsic potential to form secondary structure and that hairpin possibly is a prevailing form of miRNA in the cell [68].

### The association of structured miRNAs and the cellular pathways

**Fig 6:**
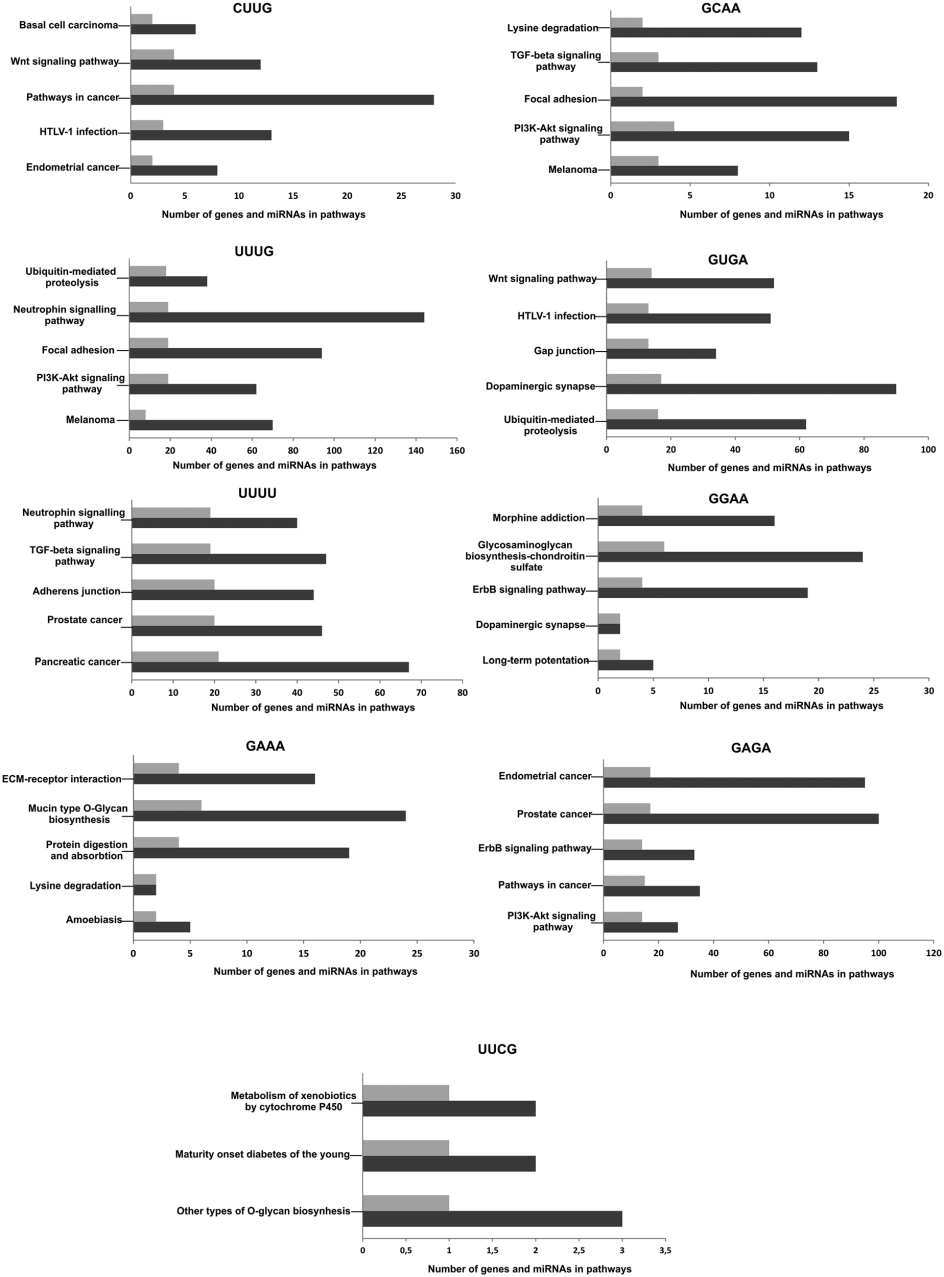
The major class of potential pathways associated with the miRNAs with the stable secondary structure and defined tetraloop. The number of genes connected to the pathways were marked with dark grey, with light grey- the number of miRNAs.

The pathway analysis revealed that miRNAs contained different sequences in the tetraloop also can cluster into specific group. This clustering approach revealed that miRNAs enriched in CUUG or GUGA sequence in the tetraloop are a regulators of the targets from Wnt signaling pathways. Neutrophin signaling pathway appears to be more associated with miRNA contained UUUG or UUUU motifs, whereas dopaminergic synapse pathways are more targeted by miRNAs with GUGA and GGAA motifs and PI3K-Akt signaling pathway by UUUG and GAGA-enriched miRNAs, respectively (Fig 6, S5 Table).

## Conclusions

For a very long time, RNA was considered to be exclusively the carrier of genetic information, but now, the group of RNAs with regulatory function have evolved into the miRNAs, long–noncoding RNAs (lncRNAs), circular RNAs or miRNA-sponges [71].

Although, one miRNA can potentially regulate hundreds of different mRNAs, the majority of transcripts are still actively expressed and translated, which supports the existence of other mechanisms, that counteract miRNA regulation, to achieve homeostasis.

Expression of the gene families or several components of a particular signaling pathway are frequently regulated by miRNAs. The pathway analysis of potential targets based on the sequence, thus the structure of miRNAs could then enhance the probability of identification and verification of the relevant miRNA-target interactions.

Our observation strongly suggest that miRNA persistence is related to biological function, thus better characterization of miRNA structure, stability and associated regulatory mechanism should provide new avenues for the characterization of their biological function. We postulate that, due to the specific sequence features, these molecules can also be involved in very well defined cellular processes depending on theirs sequence contents. Moreover, the unique features encoded in the sequence and in the structure of mature miRNA can be a key to understand the mRNA target recognition.

## Supporting Information

S1 Fig The length heterogeneity of human mature miRNAs. (DOC)

S1 File ("mirna_an.py"). Phyton scripts used for miRNA nucleotide sequences and SSR analysis. (PY)

S1 Table The results from the analysis pyrimidines/purines-rich miRNAs using PANTHER classification system. The table presents: top 10 biological processes related to input miRNAs;most significant pathways derived from overrepresentation test **and** top 10 protein classes related to input miRNAs. +/- shows over—or underrepresentation. Second and third columns contain the number of genes in reference and input list, respectively. P-value threshold is considered 0.05.
(DOC)

S2 Table Occurrence and relative count of trinucleotide repeats in mature miRNAs. (DOC)

S3 Table The results from the analysis of GU-rich miRNAs using PANTHER classification system. The table presents: top 10 biological processes related to GU-rich miRNAs; most significant pathways derived from overrepresentation test and top 10 protein classes related to GU-rich miRNAs. +/- shows over—or underrepresentation. Second and third columns contain the number of genes in reference and input list, respectively. P-value threshold is considered 0.05.
(DOC)

S4 Table The results from the analysis of interferon induction motif (IIM)- contained miRNAs using PANTHER classification system. The table presents: top 10 biological processes related to IIM motif-contained miRNAs; most significant pathways derived from overrepresentation test and top 10 protein classes related to IIM motif-contained miRNAs. +/- shows over—or underrepresentation. Second and third columns contain the number of genes in reference and input list, respectively. P-value threshold is considered 0.05.
(DOC)

S5 Table Molecular KEGG pathways analysis for miRNAs with defined tetraloops in the secondary structure. (DOC)
