# Supplementary figures and images for: The Sequence and Structure Determine the Function of Mature Human miRNAs
Source: PLoS One. 2016 Mar 31;11(3):e0151246. doi: 10.1371/journal.pone.0151246 (PMC4816427; doi:10.1371/journal.pone.0151246)

S1 Fig


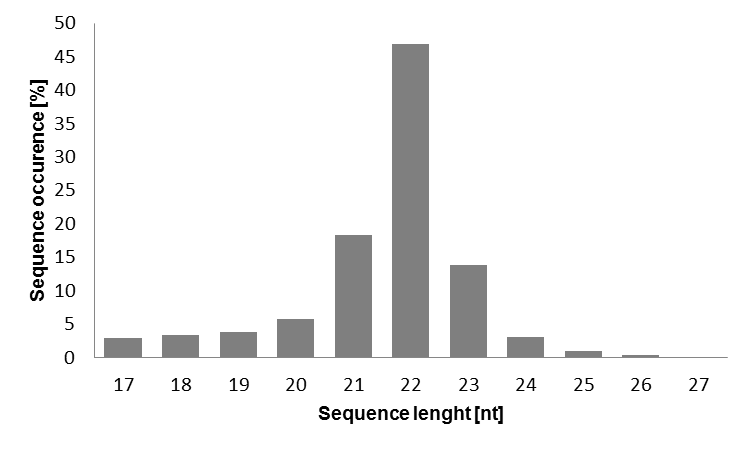

Supplement: S1 Fig — (DOC) [file pone.0151246.s001.doc]
