# Supplementary material for: The Sequence and Structure Determine the Function of Mature Human miRNAs
Source: PLoS One. 2016 Mar 31;11(3):e0151246. doi: 10.1371/journal.pone.0151246 (PMC4816427; doi:10.1371/journal.pone.0151246)
Supplement: S1 Table — The table presents: top 10 biological processes related to input miRNAs;most significant pathways derived from overrepresentation test and top 10 protein classes related to input miRNAs. +/- shows over—or underrepresentation. Second and third columns contain the number of genes in reference and input list, respectively. P-value threshold is considered 0.05. (DOC) [file pone.0151246.s003.doc]

| **GO Biological process** | ***H.sapiens* (REF) #** | **Input #** | **Fold**  **Enrichment** | **+/-** | **P-value** |
| --- | --- | --- | --- | --- | --- |
| Locomotion | 65 | 10 | >5 | + | 1.20E-03 |
| Neurotransmitter secretion | 145 | 18 | >5 | + | 6.03E-06 |
| Synaptic transmission | 331 | 24 | 3.02 | + | 5.77E-04 |
| Nervous system development | 823 | 52 | 2.63 | + | 9.35E-08 |
| Neurological system process | 1064 | 64 | 2.50 | + | 5.64E-09 |
| Protein phosphorylation | 603 | 35 | 2.42 | + | 5.05E-04 |
| Organelle organization | 571 | 33 | 2.41 | + | 1.08E-03 |
| Ectoderm development | 663 | 38 | 2.39 | + | 2.48E-04 |
| System process | 1296 | 66 | 2.12 | + | 2.16E-06 |
| Cell-cell signaling | 633 | 32 | 2.10 | + | 1.90E-02 |
| **Pathways** |  |  |  |  |  |
| PDGF signaling pathway | 138 | 18 | >5 | + | 2.01E-06 |
| Ras pathway | 79 | 10 | >5 | + | 4.37E-03 |
| Synaptic vesicle trafficking | 31 | 8 | >5 | + | 1.78E-04 |
| Angiogenesis | 154 | 18 | 4.87 | + | 1.02E-05 |
| **PANTHER protein class** |  |  |  |  |  |
| Basic helix-loop-helix transcription factor | 92 | 10 | 4.52 | + | 2.01E-02 |
| Membrane trafficing regulatory protein | 119 | 12 | 4.20 | + | 8.54E-03 |
| Membrane traffic protein | 366 | 22 | 2.50 | + | 2.17E-02 |
| Kinase | 514 | 30 | 2.43 | + | 2.22E-03 |
| Enzyme modulator | 1346 | 62 | 1.92 | + | 1.80E-04 |
| Transcription factor | 1565 | 67 | 1.78 | + | 7.54E-04 |
| Nucleic acid binding | 2297 | 90 | 1.63 | + | 5.17E-04 |

S1 Table
