# Supplementary material for: The Sequence and Structure Determine the Function of Mature Human miRNAs
Source: PLoS One. 2016 Mar 31;11(3):e0151246. doi: 10.1371/journal.pone.0151246 (PMC4816427; doi:10.1371/journal.pone.0151246)
Supplement: S2 Table — (DOC) [file pone.0151246.s004.doc]

S2 Table

| **Repaeat type** | **Number of repeats** | **Repaeat type** | **Number of repeats** |
| --- | --- | --- | --- |
| **ACA** | 0 | **GCU** | 3 |
| **ACC** | 0 | **GGA** | 6 |
| **AGA** | 3 | **GGC** | 6 |
| **AGG** | 4 | **GGU** | 0 |
| **AGU** | 0 | **GUG** | 6 |
| **AUG** | 0 | **GUU** | 0 |
| **AUU** | 0 | **UAC** | 0 |
| **CAG** | 0 | **UAU** | 0 |
| **CAU** | 0 | **UCC** | 0 |
| **CCU** | 3 | **UCG** | 0 |
| **CGU** | 0 | **UGA** | 3 |
| **CUC** | 3 | **UGC** | 0 |
| **CUG** | 0 | **UGG** | 0 |
| **CUU** | 0 | **UGU** | 0 |
| **GAG** | 0 | **UUC** | 0 |
| **GAU** | 3 | **UUG** | 0 |
| **GCC** | 3 |  | |
| **Total** 43 | | | |
