# Supplementary material for: The Sequence and Structure Determine the Function of Mature Human miRNAs
Source: PLoS One. 2016 Mar 31;11(3):e0151246. doi: 10.1371/journal.pone.0151246 (PMC4816427; doi:10.1371/journal.pone.0151246)
Supplement: S3 Table — The table presents: top 10 biological processes related to GU-rich miRNAs; most significant pathways derived from overrepresentation test and top 10 protein classes related to GU-rich miRNAs. +/- shows over—or underrepresentation. Second and third columns contain the number of genes in reference and input list, respectively. P-value threshold is considered 0.05. (DOC) [file pone.0151246.s005.doc]

S3 Table

| **GO Biological process** | **H.sapiens (REF) #** | **Input #** | **Fold**  **Enrichmnet** | **+/-** | **P-value** |
| --- | --- | --- | --- | --- | --- |
| mRNA 3’-end processing | 30 | 34 | 2.47 | + | 6.64E-04 |
| Embryo development | 150 | 134 | 1.95 | + | 4.33E-10 |
| mRNA transcription | 57 | 47 | 1.80 | + | 3.33E-02 |
| Protein targeting | 112 | 89 | 1.73 | + | 2.58E-04 |
| Protein localization | 116 | 89 | 1.67 | + | 9.86E-04 |
| Negative regulation of apoptotic process | 95 | 71 | 1.63 | + | 1.82E-02 |
| Nervous system development | 823 | 601 | 1.59 | + | 3.63E-25 |
| Muscle organ development | 288 | 200 | 1.51 | + | 4.30E-06 |
| Cellular protein modification process | 1317 | 909 | 1.50 | + | 7.50E-31 |
| Catabolic process | 407 | 277 | 1.48 | + | 6.27E-08 |
| Synaptic transmission | 331 | 215 | 1.41 | + | 1.50E-04 |
| **Pathways** |  |  |  |  |  |
| Opioid proopiomelanocortin pathway | 20 | 24 | 2.61 | + | 5.08E-03 |
| Metabotropic glutamate receptor group II pathway | 34 | 40 | 2.56 | + | 2.65E-05 |
| Opioid proenkephalin pathway | 21 | 24 | 2.49 | + | 1.07E-02 |
| Ionotropic | 57 | 63 | 2.41 | + | 1.09E-07 |
| PI3 kinase pathway | 49 | 54 | 2.40 | + | 1.86E-06 |
| Hedgehog signaling pathway | 22 | 24 | 2.38 | + | 2.12E-02 |
| Insulin/IGF pathway-mitogen activated protein kinase kinase/MAP kinase cascade | 33 | 36 | 2.38 | + | 5.57E-04 |
| Axon guidance mediated by netrin | 34 | 37 | 2.37 | + | 4.35E-04 |
| p38 MAPK pathway | 39 | 41 | 2.29 | + | 3.04E-04 |
| 5HT1 type receptor mediated signaling pathway | 31 | 32 | 2.25 | + | 5.27E-03 |
| **PANTHER protein class** |  |  |  |  |  |
| Serine/threonine protein kinase receptor | 19 | 24 | 2.75 | + | 3.01E-03 |
| Protein kinase receptor | 33 | 38 | 2.51 | + | 1.15E-04 |
| SNARE protein | 42 | 44 | 2.28 | + | 1.90E-04 |
| Voltage-gated sodium channnel | 36 | 36 | 2.18 | + | 4.53E-03 |
| Sodium channel | 36 | 36 | 2.18 | + | 4.53E-03 |
| Ubiquitin-protein ligase | 189 | 163 | 1.88 | + | 2.91E-11 |
| Protein kinase | 373 | 224 | 1.73 | + | 1.81E-16 |
| G- protein | 211 | 168 | 1.73 | + | 5.77E-09 |
| Small GTPase | 133 | 104 | 1.70 | + | 6.65E-05 |
| Basic helix-loop-helix transcription factor | 92 | 71 | 1.68 | + | 6.48E-03 |
