# Supplementary material for: The Sequence and Structure Determine the Function of Mature Human miRNAs
Source: PLoS One. 2016 Mar 31;11(3):e0151246. doi: 10.1371/journal.pone.0151246 (PMC4816427; doi:10.1371/journal.pone.0151246)
Supplement: S4 Table — The table presents: top 10 biological processes related to IIM motif-contained miRNAs; most significant pathways derived from overrepresentation test and top 10 protein classes related to IIM motif-contained miRNAs. +/- shows over—or underrepresentation. Second and third columns contain the number of genes in reference and input list, respectively. P-value threshold is considered 0.05. (DOC) [file pone.0151246.s006.doc]

| **GO Biological process** | **H.sapiens (REF) #** | **Input #** | **Fold**  **Enrichment** | **+/-** | **P-value** |
| --- | --- | --- | --- | --- | --- |
| mRNA 3’-end processing | 30 | 17 | 2.77 | + | 4.91E-02 |
| Embryo development | 150 | 73 | 2.38 | + | 1.11E-08 |
| Protein targeting | 112 | 48 | 2.10 | + | 6.51E-04 |
| Protein phosphorylation | 603 | 242 | 1.96 | + | 9.82E-20 |
| mRNA splicing | 183 | 69 | 1.84 | + | 4.75E-04 |
| Angiogenesis | 198 | 73 | 1.80 | + | 5.32E-04 |
| Induction of apoptosis | 129 | 47 | 1.78 | + | 3.86E-02 |
| Nervous system development | 823 | 290 | 1.72 | + | 4.05E-16 |
| Immune system process | 1391 | 224 | 1.69 | + | 1.59E-02 |
| Immune response | 518 | 61 | 1.58 | + | 2.60E-04 |
| Macrophage activation | 167 | 11 | 1.32 | + | 8.18E-04 |
| **Pathways** |  |  |  |  |  |
| PI3 kinase pathway | 49 | 38 | 3.79 | + | 1.73E-09 |
| Hedgehog signaling pathway | 22 | 16 | 3.56 | + | 3.07E-03 |
| Axon guidance mediated by semaphorins | 22 | 15 | 3.33 | + | 1.12E-02 |
| p38 MAPK pathway | 39 | 26 | 3.26 | + | 4.97E-05 |
| Insulin/IGF pathway-mitogen activated protein kinase kinase/MAP kinase cascade | 33 | 20 | 2.96 | + | 4.07E-03 |
| Oxidative stress response | 27 | 16 | 2.90 | + | 3.17E-02 |
| Interferon-gamma signaling pathway | 39 | 22 | 2.75 | + | 2.49E-02 |
| EGF receptor signaling pathway | 130 | 72 | 2.71 | + | 3.28E-11 |
| FGF signaling pathway | 123 | 68 | 2.70 | + | 1.62E-10 |
| Ras pathway | 79 | 34 | 2.10 | + | 1.07E-02 |
| **PANTHER protein class** |  |  |  |  |  |
| TGF-beta receptor | 15 | 12 | 3.91 | + | 1.76E-02 |
| mRNA polyadenylation factor | 21 | 14 | 3.26 | + | 3.17E-02 |
| Protein kinase receptor | 33 | 18 | 2.67 | + | 4.72E-02 |
| HMG box transcription factor | 45 | 22 | 2.39 | + | 4.70E-02 |
| Non –receptor serine/ threonine protein kinase | 272 | 124 | 2.22 | + | 2.47E-13 |
| Kinase modulator | 194 | 86 | 2.17 | + | 2.05E-08 |
| Protein kinase | 373 | 165 | 2.16 | + | 7.80E-17 |
| Chromatin/chromatin-binding protein | 184 | 80 | 2.13 | + | 2.11E-07 |
| Kinase inhibitor | 73 | 31 | 2.08 | + | 3.58E-02 |
| G-protein | 211 | 78 | 1.81 | + | 2.06E-04 |

S4 Table
