# Supplementary material for: The Sequence and Structure Determine the Function of Mature Human miRNAs
Source: PLoS One. 2016 Mar 31;11(3):e0151246. doi: 10.1371/journal.pone.0151246 (PMC4816427; doi:10.1371/journal.pone.0151246)
Supplement: S5 Table — (DOC) [file pone.0151246.s007.doc]

| **Motif in a loop** | **KEGG pathway(pathawy ID)** | **p-value**  **(union)** |
| --- | --- | --- |
| **CUUG** | - Basal cell carcinoma (hsa05127) - Wnt signaling pathway (hsa04310) - Pathways in cancer (hsa05200) - HTLV-1 infection (hsa05166) - Endometrial cancer (hsa05123) | 0.0002603625  0.0003335464  0.001969395  0.002748364  0.01658278 |
| **UUUG** | - Ubiquitin-mediated proteolysis (hsa04120) - Neurotrophin signalling pathway (hsa04722) - Focal adhesion (hsa04510) - PI3K-Akt signaling pathway (hsa04151) - Melanoma (hsa05218) | 1.09105E-33  6.568774E-28  6.665626E-27  1.049143E-24  1.164063E-18 |
| **GCAA** | - Lysine degradation (hsa00310) - TGF-beta signaling pathway (hsa04350) - Focal adhesion (hsa04510) - Ubiquitin-mediated proteolysis (hsa04120) - Wnt signaling pathway (hsa04310) | 3.156403E-09  2.637396E-06  0.0005666272  0.001157976  0.001927753 |
| **GUGA** | - Wnt signaling pathway (hsa04310) - HTLV-1 infection (hsa05166) - Gap junction (hsa04540) - Dopaminergic synapse (hsa04728) - Ubiquitin-mediated proteolysis (hsa04120) | 9.005047E-13  5.368155E-10  6.428566E-10  6.428566E-10  7.76398E-10 |
| **UUUU** | - Neurotrophin signalling pathway (hsa04722) - TGF-beta signaling pathway (hsa04350) - Adherens junction (hsa04350) - Prostate cancer (hsa05215) - Pancreatic cancer (hsa05212) | 9.533143E-35  9.885926E-28  1.509373E-25  1.270366E-24  1.286761E-22 |
| **GGAA** | - Morphine addiction (hsa05032) - Glycosaminoglycan biosynthesis-chondroitin sulfate (hsa00532) - ErbB signaling pathway (hsa04012) - Dopaminergic synapse (hsa04728) - Long-term potentiation (hsa04720) | 3.582207E-06  5.061091E-06  9.824829E-06  0.0002367772  0.001532743 |
| **GAAA** | - ECM-receptor interaction (hsa04512) - Mucin type O-Glycan biosynthesis (hsa00512) - Protein digestion and absorption (hsa04974) - Lysine degradation (hsa00310) - Amoebiasis (hsa05146) | <1E-16  <1E-16  4.440892E-16  4.440892E-16  1.287859E-14 |
| **UUCG** | - Metabolism of xenobiotics by cytochrome P450 (hsa00980) - Maturity onset diabetes of the young (hsa04950) - Other types of O-glycan biosynthesis (hsa00514) | 4.778008E-09  0.00798776  0.04455185 |
| **GAGA** | - Endometrial cancer (hsa05123) - Prostate cancer (hsa05215) - ErbB signaling pathway (hsa04012) - Pathways in cancer (hsa200) - PI3K-Akt signaling pathway (hsa04151) | 5.004043E-25  8.307837E-17  1.206676E-15  2.729161E-15  1.464602E-14 |

S5 Table
